# Hepatic Resection Is Associated With Improved Long-Term Survival Compared to Radio-Frequency Ablation in Patients With Multifocal Hepatocellular Carcinoma

**DOI:** 10.3389/fonc.2020.00110

**Published:** 2020-02-11

**Authors:** Yang-yang Yue, Wei-li Zhou

**Affiliations:** ^1^Department of Health Management, Sheng-Jing Hospital of China Medical University, Shenyang, China; ^2^Department of General Surgery, Sheng-Jing Hospital of China Medical University, Shenyang, China

**Keywords:** hepatic resection, radio-frequency ablation, hepatocellular carcinoma, liver cancer, multifocal tumor

## Abstract

**Background:** The prognosis of patients with hepatocellular carcinoma (HCC) is of major public health interest. However, studies comparing hepatic resection (HR) and radio-frequency ablation (RFA) applied to multifocal HCC are limited. This study aimed to compare the efficacies of HR and RFA in patients with multifocal HCC.

**Methods:** We retrospectively analyzed a cohort from the Surveillance, Epidemiology, and End Results database between 2004 and 2015. Disease-specific survival and overall survival rates were assessed before and after propensity score matching (PSM).

**Results:** In total, 2,201 patients with multifocal HCC treated with HR (*n* = 1,095) or RFA (*n* = 1,106) were included; 1,096 patients were identified after nearest-neighbor PSM at a ratio of 1:1 (HR: *n* = 548; RFA: *n* = 548). In the multivariate Cox regression model, HR was associated with significantly improved disease-specific survival [before PSM: hazard ratio 0.67, 95% confidence interval (CI) 0.57–0.79, *p* < 0.001; after PSM: hazard ratio 0.69, 95% CI 0.58–0.82, *p* < 0.001] and overall survival (before PSM: hazard ratio 0.67, 95% CI 0.58–0.78, *p* < 0.001; after PSM: hazard ratio 0.69, 95% CI 0.59–0.80, *p* < 0.001) compared to RFA in patients with multifocal HCC. In the survival curve analysis, the disease-specific survival of the HR group was similar to that of the RFA group before PSM (*p* = 0.936, log-rank test) but was significantly longer after PSM (*p* < 0.001) in all patients. Multivariate analyses revealed that differentiation grade, alpha-fetoprotein, tumor size, and tumor extension were independent predictors of poor prognosis in patients with multifocal HCC.

**Conclusions:** The long-term survival rate of HR is better than that of RFA in patients with multifocal HCC. HR may serve as a first-line treatment for patients with multifocal HCC. The presence of large tumors and vascular invasion are not contraindications for HR.

## Introduction

According to global cancer statistics in 2018, hepatocellular carcinoma (HCC) is the sixth most prevalent cancer and the fourth primary cause of cancer-related mortality with more than 841,000 newly diagnosed cases, accounting for 5.7% of all cancer patients, and 781,000 deaths annually, accounting for 8.2% of all cancer-related deaths (1, 2).

According to the Barcelona Clinic for Liver Cancer (BCLC) staging system which is endorsed by the American Association for the Study of Liver Disease, European Association for the Study of Liver Disease, and European Society for Medical Oncology, HCC patients with BCLC very-early-stage (stage 0, single tumor <2 cm in size) and early-stage (stage A, no more than three tumors each <3 cm in size) carcinoma and without major vascular invasion or extrahepatic metastasis are recommended to undergo hepatic resection (HR), liver transplantation, or radio-frequency ablation (RFA). However, for patients with BCLC intermediate-stage (stage B) and advanced-stage (stage C) disease, trans-arterial chemoembolization is the first-line therapy; therefore, HR, liver transplantation and RFA may not be performed (3–5).

Recently, several studies have reported that HR is more favorable than RFA and trans-arterial chemoembolization regardless of BCLC stage, which expands the application of HR to intermediate-stage (stage B) HCC (6–10). Moreover, a recent study has proven that HR plus RFA resulted in better long-term survival than trans-arterial chemoembolization in patients with multifocal HCC with tumors <5 cm or >5 cm in size; however, their sample size was very small (11). Another study found that HR plus RFA were superior to HR alone regarding the outcome of HCC patients; however, only 73 subjects were included (12). Few studies have directly compared the efficacies of HR and RFA in multifocal HCC tumors; moreover, some studies have shown that HR is similar to RFA, while others have suggested that HR is superior to RFA in multifocal tumors <=3 cm in size (13, 14). In addition, few studies have focused on the differences in the outcomes of HR and RFA in multifocal tumors sized 3–5 cm, and no study has compared the efficacy of HR with that of RFA in multifocal tumors >5 cm in size, especially in tumors with extrahepatic metastasis and vascular invasion.

Using the Surveillance, Epidemiology, and End Results (SEER) database, the biggest cancer surveillance program that covers ~34.6% of the population in the United States, we conducted a comprehensive comparison of the efficacies of HR and RFA applied to multifocal HCC tumors of any size using a large real-world sample. The primary aim of the study was to compare the effectiveness of HR and RFA in patients with multifocal HCC, and the secondary aim was to assess confounding factors influencing survival outcomes.

## Materials and Methods

### Study Population

We obtained a case listing of patients with liver cancer from the SEER program of the National Cancer Institute between 2004 and 2015. Patients were filtered using inclusion criteria as follows: (1) patients with a primary site code C22.0 and International Classification of Diseases for Oncology, Third Edition histology codes 8170-8175, (2) SEER Collaborative Stage (CS) Extension Codes 390, 400, 420, 440, 630, and 635 for multifocal tumors, and (3) SEER Surgery of Primary Site Codes 20–26, 30, 36–38, 50–52, and 59–60 for HR and 16 for RFA. RFA also includes microwave ablation. We excluded samples based on the following criteria: (1) the presence of more than one primary cancer, (2) 0 months of survival, and (3) younger than 18 years old. All patients in this study were collected from the SEER database, and we signed the “Data-use Agreement for the SEER 1973–2015 Research Data File” and received permission.

The primary outcome was disease-specific survival (DSS), which was the time until death attributed to HCC. The secondary outcome was overall survival (OS), which was the time until death caused by any disease. Variables in the analysis were age, marital status, gender, race, tumor differentiation grade, lymph nodes, distant metastasis, radiotherapy, chemotherapy, alpha-fetoprotein (AFP) level, fibrosis score, tumor size, and tumor extension. Tumor extension indicated the extent of contiguous growth of the primary tumor within the liver or its direct extension into neighboring organs. The meaning of tumor extension are as follows: 390, confined to one lobe without intrahepatic vascular invasion (IVI); 400, confined to one lobe with IVI; 420, extension to gallbladder with or without IVI; 440, extension to multiple lobes or on surface of parenchyma; 630, confined to one lobe with major vascular invasion (MVI); 635, extension to multiple lobes or on surface of liver parenchyma, with MVI.

We categorized patients by tumor size using 2, 3, and 5 cm as cut-off values. We selected these cut-offs, because some guidelines maintain that RFA is the first-line therapy rather than HR for tumors sized <2 cm (BCLC stage 0) and <3 cm (BCLC stage A) (3–5, 15). Additionally, some studies insist that RFA is more beneficial for tumors <5 cm in diameter, while others argue that HR is preferable to RFA for tumors 2–5 cm in size (13, 16). Moreover, we also wanted to compare the efficacies of HR and RFA for multifocal tumors >5 cm in size.

### Statistical Analyses

We used nearest-neighbor propensity score matching (PSM) at a ratio of 1:1 and dropped 50 percent of the HR observations at which the propensity score density of the RFA observations is the lowest by applying “psmatch2” command with the option of “common trim(50).” Finally, all the variables achieved a complete balance between the HR and RFA groups.

All statistical analyses and figure rendering were carried out using STATA software, version 15 (StataCorp, College Station, Texas). The demographic and clinical characteristics between HR and RFA cases were compared using Pearson's chi-square test. Survival curves were generated using the Kaplan-Meier method, and the significance of the differences in survival rates was examined using the log-rank test. We carried out multivariate Cox proportional hazards regression to explore the efficacy of the HR and RFA, and control for confounding factors correlating to DSS and OS. Corresponding hazard ratios and 95% confidence intervals (CIs) were estimated from the Cox model, too. A two-tailed *p*-value < 0.05 was considered statistically significant.

## Results

### Baseline Demographic and Clinical Characteristics

**Figure 1:**
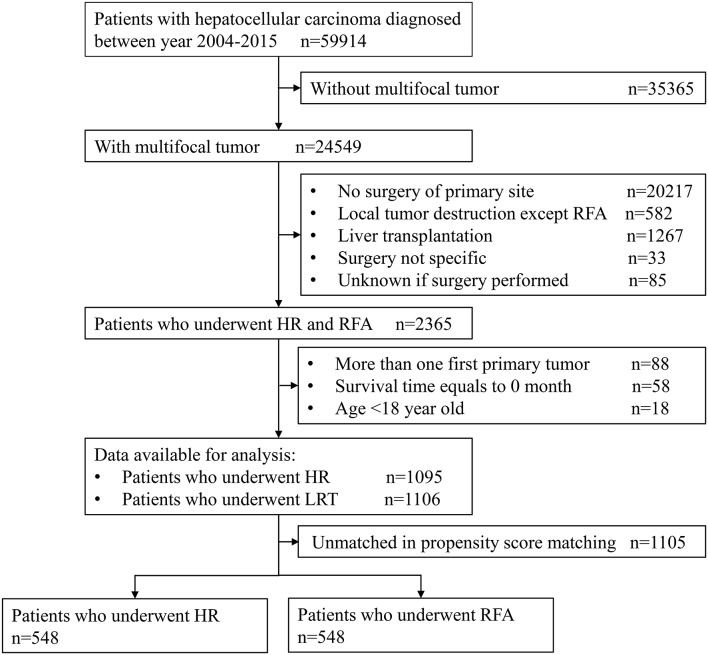
Flow chart of the sample selection procedure.

The sample selection procedure was illustrated in Figure 1. Of the 59,914 patients with HCC between 2004 and 2015, we obtained 2,201 patients meeting inclusion criteria for final analysis. Of them, 1,106 cases underwent RFA and 1,095 underwent HR; after PSM, there were both 548 cases in RFA and HR groups.

The baseline demographic and clinical data are presented in Table 1. Before PSM, the RFA group contained more young patients; had a higher proportion of divorced, widowed, or separated persons; had a higher percentage of men, white people, and patients with unknown tumor differentiation grade; had a higher level of alpha-fetoprotein; were more likely to have liver cirrhosis and smaller tumor size; more likely to undergo chemotherapy; and had a higher proportion of people with tumor extension 390 and 440 than the HR group. After PSM, all variables were completely balanced between the HR and RFA groups.

**Table 1:** Baseline demographic and clinical characteristics before and after propensity score matching.

**Characteristics**	**Before PSM**			**After PSM**		
	**RFA** **(*n* = 1,106)**	**HR** **(*n* = 1,095)**	***P* value**	**RFA** **(*n* = 548)**	**HR** **(*n* = 548)**	***P* value**
**Age (years)**			0.034			0.950
18–65	719 (65.01)	664 (60.64)		341 (62.23)	342 (62.41)	
>65	387 (34.99)	431 (39.36)		207 (37.77)	206 (37.59)	
**Marital status**			0.016			0.889
Divorced, widowed, and separated	250 (22.60)	200 (18.26)		107 (19.53)	113 (20.62)	
Married	600 (54.25)	663 (60.55)		319 (58.21)	308 (56.20)	
Single and unmarried	215 (19.44)	200 (18.26)		106 (19.34)	108 (19.71)	
Unknown	41 (3.71)	32 (2.92)		16 (2.92)	19 (3.47)	
**Gender**			0.009			0.776
Female	247 (22.33)	297 (27.12)		132 (24.09)	128 (23.36)	
Male	859 (77.67)	798 (72.88)		416 (75.91)	420 (76.64)	
**Race**			<0.001			0.873
Black	135 (12.21)	160 (14.61)		75 (13.69)	80 (14.6)	
Other	195 (17.63)	296 (27.03)		113 (20.62)	110 (20.07)	
Unknown	6 (0.54)	7 (0.64)		5 (0.91)	3 (0.55)	
White	770 (69.62)	632 (57.72)		355 (64.78)	355 (64.78)	
**Grade**			<0.001			0.111
Well	163 (14.74)	154 (14.06)		116 (21.17)	117 (21.35)	
Moderate	209 (18.90)	510 (46.58)		197 (35.95)	220 (40.15)	
Poor	43 (3.89)	254 (23.20)		43 (7.85)	57 (10.40)	
Undifferentiated/anaplastic	4 (0.36)	21 (1.92)		4 (0.73)	4 (0.73)	
Unknown	687 (62.12)	156 (14.25)		188 (34.31)	150 (27.37)	
**Lymph nodes**			0.568			0.949
N0	1028 (92.95)	1020 (93.15)		510 (93.07)	508 (92.70)	
N1	35 (3.16)	40 (3.65)		19 (3.47)	19 (3.47)	
NX	43 (3.89)	35 (3.20)		19 (3.47)	21 (3.83)	
**Distant metastasis**			0.132			0.773
M0	1071 (96.84)	1042 (95.16)		525 (95.80)	520 (94.89)	
M1	25 (2.26)	37 (3.38)		18 (3.28)	22 (4.01)	
MX	10 (0.90)	16 (1.46)		5 (0.91)	6 (1.09)	
**Radiation**			0.194			0.589
No	1051 (95.03)	1053 (96.16)		517 (94.34)	521 (95.07)	
Yes	55 (4.97)	42 (3.84)		31 (5.66)	27 (4.93)	
**Chemotherapy**			<0.001			0.337
No	647 (58.50)	819 (74.79)		357 (65.15)	372 (67.88)	
Yes	459 (41.50)	276 (25.21)		191 (34.85)	176 (32.12)	
**AFP**			<0.001			0.692
Within normal limit	233 (21.07)	261 (23.84)		132 (24.09)	134 (24.45)	
Positive/elevate	718 (64.92)	580 (52.97)		319 (58.21)	307 (56.02)	
Unknown	155 (14.01)	254 (23.20)		97 (17.70)	107 (19.53)	
**Fibrosis score**			<0.001			0.507
0–4	42 (3.80)	188 (17.17)		32 (5.84)	34 (6.20)	
5–6	383 (34.63)	188 (17.17)		154 (28.10)	137 (25.00)	
Unknown	681 (61.57)	719 (65.66)		362 (66.06)	377 (68.80)	
**Tumor size (cm)**			<0.001			0.243
0–2	155 (14.01)	62 (5.66)		63 (11.50)	59 (10.77)	
2–3	368 (33.27)	110 (10.05)		121 (22.08)	100 (18.25)	
3–5	401 (36.26)	236 (21.55)		199 (36.31)	197 (35.95)	
>5	182 (16.46)	687 (62.74)		165 (30.11)	192 (35.04)	
**Tumor extension**			<0.001			0.823
390	546 (49.37)	430 (39.27)		276 (50.36)	268 (48.91)	
400	44 (3.98)	164 (14.98)		34 (6.20)	39 (7.12)	
420	4 (0.36)	11 (1.00)		3 (0.55)	4 (0.73)	
440	448 (40.51)	276 (25.21)		191 (34.85)	182 (33.21)	
630	46 (4.16)	180 (16.44)		35 (6.39)	42 (7.66)	
635	18 (1.63)	34 (3.11)		9 (1.64)	13 (2.37)	

*PSM, propensity score matching; RFA, radio-frequency ablation; HR, hepatic resection; AFP, alpha-fetoprotein; 390, confined to one lobe without intrahepatic vascular invasion (IVI); 400, confined to one lobe with IVI; 420, extension to gallbladder with or without IVI; 440, extension to multiple lobes or on surface of parenchyma; 630, confined to one lobe with major vascular invasion (MVI); 635, extension to multiple lobes or on surface of liver parenchyma, with MVI*.

### Multivariate Cox Proportional Hazards Regression of Disease-Specific Survival and Overall Survival

In the Cox analysis of DSS, before PSM, patients who underwent HR had a significantly longer survival outcome than did those who underwent RFA (hazard ratio 0.67, 95% CI 0.57–0.79, *p* < 0.001). Lower tumor differentiation grades, higher alpha-fetoprotein level, bigger tumor size, and higher tumor extension were all significantly correlated with poor prognosis. After PSM, HR was still associated with prolonged survival (hazard ratio 0.69, 95% CI 0.58–0.82, *p* < 0.001) compared to RFA. Lower tumor differentiation grades, higher alpha-fetoprotein level, bigger tumor size, and higher tumor extension remained significantly associated with worse survival (Table 2).

**Table 2:** Multivariate Cox proportional hazards regression for disease-specific survival before and after propensity score matching.

**Characteristics**	**Before PSM**			**After PSM**		
	**Hazard ratio**	**95% CI**	***P* value**	**Hazard ratio**	**95% CI**	***P* value**
**Treatment**						
RFA	Reference			Reference		
HR	0.67	0.57–0.79	<0.001	0.69	0.58–0.82	<0.001
**Age (years)**						
18–65	Reference			Reference		
>65	1.07	0.93–1.22	0.340	0.98	0.81–1.19	0.842
**Marital status**						
Divorced, widowed, and separated	Reference			Reference		
Married	0.90	0.77–1.06	0.215	0.93	0.73–1.17	0.526
Single and unmarried	1.00	0.82–1.22	0.992	0.92	0.68–1.23	0.552
Unknown	0.70	0.50–0.98	0.036	0.65	0.39–1.08	0.097
**Gender**						
Female	Reference			Reference		
Male	1.07	0.93–1.24	0.331	0.98	0.79–1.21	0.838
**Race**						
Black	Reference			Reference		
Other	0.89	0.72–1.10	0.277	0.89	0.65–1.22	0.484
Unknown	0.44	0.092–2.09	0.301	0.31	0.037–2.62	0.283
White	1.03	0.86–1.23	0.742	0.97	0.75–1.27	0.849
**Grade**						
Well	Reference			Reference		
Moderate	1.36	1.10–1.68	0.005	1.49	1.15–1.93	0.003
Poor	1.98	1.54–2.54	<0.001	1.99	1.40–2.83	<0.001
Undifferentiated/anaplastic	2.95	1.48–5.87	0.002	2.05	0.61–6.88	0.244
Unknown	1.40	1.14–1.72	0.001	1.46	1.11–1.92	0.006
**Lymph nodes**						
N0	Reference			Reference		
N1	1.21	0.87–1.67	0.255	0.88	0.53–1.46	0.625
NX	0.96	0.67–1.38	0.831	0.80	0.45–1.40	0.431
**Distant metastasis**						
M0	Reference			Reference		
M1	1.50	1.01–2.22	0.045	1.56	0.93–2.61	0.094
MX	1.18	0.66–2.13	0.574	1.34	0.47–3.80	0.586
**Radiotherapy**						
No	Reference			Reference		
Yes	1.16	0.88–1.52	0.290	1.14	0.79–1.66	0.484
**Chemotherapy**						
No	Reference			Reference		
Yes	0.94	0.83–1.08	0.389	0.91	0.76–1.10	0.349
**AFP**						
Within normal limit	Reference			Reference		
Positive/elevated	1.37	1.17–1.60	<0.001	1.33	1.07–1.66	0.011
Unknown	1.39	1.14–1.70	0.001	1.51	1.15–2.00	0.004
**Fibrosis score**						
0–4	Reference			Reference		
5–6	1.15	0.92–1.44	0.228	1.11	0.79–1.56	0.557
Unknown	1.10	0.90–1.36	0.348	1.11	0.80–1.53	0.527
**Tumor size (cm)**						
0–2	Reference			Reference		
2–3	1.46	1.10–1.93	0.008	1.71	1.13–2.61	0.012
3–5	2.05	1.56–2.70	<0.001	2.18	1.47–3.24	<0.001
>5	2.98	2.26–3.93	<0.001	3.24	2.17–4.84	<0.001
**Tumor extension**						
390	Reference			Reference		
400	1.21	0.96–1.53	0.104	1.20	0.83–1.76	0.334
420	1.05	0.49–2.22	0.906	1.54	0.58–4.09	0.382
440	1.32	1.15–1.52	<0.001	1.47	1.21–1.79	<0.001
630	1.51	1.22–1.88	<0.001	1.78	1.22–2.58	0.003
635	1.97	1.32–2.93	0.001	1.81	0.90–3.64	0.097

*PSM, propensity score matching; CI, confidence interval; RFA, radio-frequency ablation; HR, hepatic resection; AFP, alpha-fetoprotein; 390, confined to one lobe without intrahepatic vascular invasion (IVI); 400, confined to one lobe with IVI; 420, extension to gallbladder with or without IVI; 440, extension to multiple lobes or on surface of parenchyma; 630, confined to one lobe with major vascular invasion (MVI); 635, extension to multiple lobes or on surface of liver parenchyma, with MVI*.

In the Cox analysis of OS, before PSM, patients in the HR group had significantly improved survival than those in the RFA group (hazard ratio 0.67, 95% CI 0.58–0.78, *p* < 0.001). Similarly, after PSM, patients treated with HR had a better prognosis than did those treated with RFA (hazard ratio 0.69, 95% CI 0.59–0.80, *p* < 0.001). Moreover, tumor differentiation grade, alpha-fetoprotein level, tumor size, and tumor extension were all significantly associated with survival both before and after PSM (Table 3).

**Table 3:** Multivariate Cox proportional hazards regression for overall survival before and after propensity score matching.

**Characteristics**	**Before PSM**			**After PSM**		
	**Hazard ratio**	**95% CI**	***P* value**	**Hazard ratio**	**95% CI**	***P* value**
**Treatment**						
RFA	Reference			Reference		
HR	0.67	0.58–0.78	<0.001	0.69	0.59–0.80	<0.001
**Age (years)**						
18–65	1			1		
>65	1.09	0.97–1.23	0.139	1.02	0.86–1.22	0.786
**Marital status**						
Divorced, widowed, and separated	Reference			Reference		
Married	0.87	0.75–1.00	0.054	0.89	0.73–1.10	0.286
Single and unmarried	0.94	0.78–1.12	0.467	0.84	0.65–1.09	0.187
Unknown	0.67	0.50–0.89	0.007	0.68	0.44–1.05	0.085
**Gender**						
Female	Reference			Reference		
Male	1.15	1.01–1.31	0.038	1.06	0.88–1.29	0.547
**Race**						
Black	Reference			Reference		
Other	0.84	0.69–1.02	0.080	0.81	0.60–1.07	0.138
Unknown	0.54	0.14–2.02	0.361	0.24	0.031–1.93	0.181
White	1.04	0.88–1.23	0.631	0.97	0.76–1.23	0.775
**Grade**						
Well	Reference			Reference		
Moderate	1.31	1.08–1.58	0.005	1.45	1.15–1.82	0.002
Poor	1.84	1.47–2.31	<0.001	1.95	1.43–2.64	<0.001
Undifferentiated/anaplastic	2.70	1.40–5.19	0.003	1.87	0.53–6.65	0.332
Unknown	1.39	1.16–1.67	<0.001	1.50	1.17–1.91	0.001
**Lymph nodes**						
N0	Reference			Reference		
N1	1.14	0.85–1.54	0.382	0.87	0.55–1.37	0.550
NX	0.98	0.72–1.34	0.903	0.80	0.47–1.36	0.414
**Distant metastasis**						
M0	Reference			Reference		
M1	1.42	0.98–2.05	0.067	1.46	0.92–2.31	0.109
MX	1.22	0.72–2.04	0.459	1.21	0.42–3.47	0.723
**Radiotherapy**						
No	Reference			Reference		
Yes	1.12	0.87–1.43	0.372	1.17	0.83–1.63	0.368
**Chemotherapy**						
No	Reference			Reference		
Yes	0.91	0.81–1.02	0.095	0.85	0.72–1.00	0.054
**AFP**						
Within normal limit	Reference			Reference		
Positive/elevated	1.31	1.14–1.50	<0.001	1.21	1.00–1.47	0.051
Unknown	1.27	1.06–1.51	0.009	1.31	1.02–1.68	0.035
**Fibrosis score**						
0–4	Reference			Reference		
5–6	1.12	0.91–1.38	0.283	1.08	0.79–1.47	0.621
Unknown	1.12	0.93–1.36	0.238	1.15	0.86–1.54	0.334
**Tumor size (cm)**						
0–2	Reference			Reference		
2–3	1.27	1.02–1.59	0.036	1.36	0.98–1.90	0.068
3–5	1.59	1.28–1.99	<0.001	1.55	1.13–2.13	0.006
>5	2.20	1.75–2.76	<0.001	2.25	1.63–3.09	<0.001
**Tumor extension**						
390	Reference			Reference		
400	1.29	1.05–1.59	0.014	1.31	0.95–1.79	0.096
420	1.05	0.53–2.10	0.887	1.83	0.86–3.90	0.115
440	1.32	1.17–1.50	<0.001	1.40	1.17–1.67	<0.001
630	1.59	1.31–1.94	<0.001	1.69	1.21–2.36	0.002
635	2.09	1.45–3.01	<0.001	2.18	1.21–3.96	0.010

*PSM, propensity score matching; CI, confidence interval; RFA, radio-frequency ablation; HR, hepatic resection; AFP, alpha-fetoprotein; 390, confined to one lobe without intrahepatic vascular invasion (IVI); 400, confined to one lobe with IVI; 420, extension to gallbladder with or without IVI; 440, extension to multiple lobes or on surface of parenchyma; 630, confined to one lobe with major vascular invasion (MVI); 635, extension to multiple lobes or on surface of liver parenchyma, with MVI*.

### Survival Curve Analysis of Disease-Specific Survival

**Figure 2:**
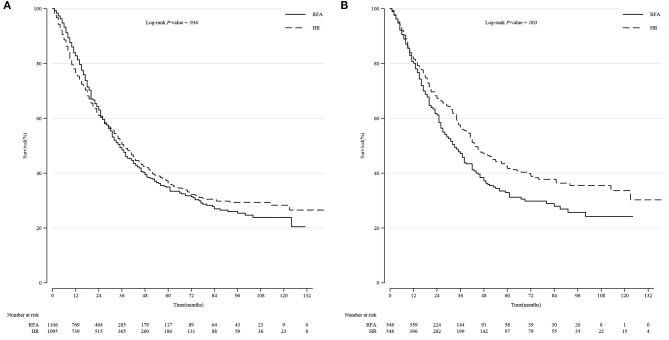
Comparison of Kaplan-Meier survival curves of DSS between the HR and RFA groups before and after PSM in all patients. DSS was similar between the HR and RFA groups before PSM **(A)** but significantly different after PSM **(B)**. DSS, disease-specific survival; HR, hepatic resection; RFA, radio-frequency ablation; PSM, propensity score matching.

Among all the patients, the long-term survival rate of the HR group was not significantly different from that of the RFA group (*p* = 0.936, log-rank test). However, after PSM, we observed a significant improvement in the survival rate in patients treated with HR (*p* = 0.003) (Figure 2).

### Subgroup Survival Curve Analysis of Disease-Specific Survival After Propensity Score Matching

Because the effect size of HR was very similar for DSS and OS, we only analyzed the DSS survival curve. We conducted a Kaplan-Meier survival curve analysis and log-rank test stratified by tumor differentiation grade, tumor size, and extension.

**Figure 3:**
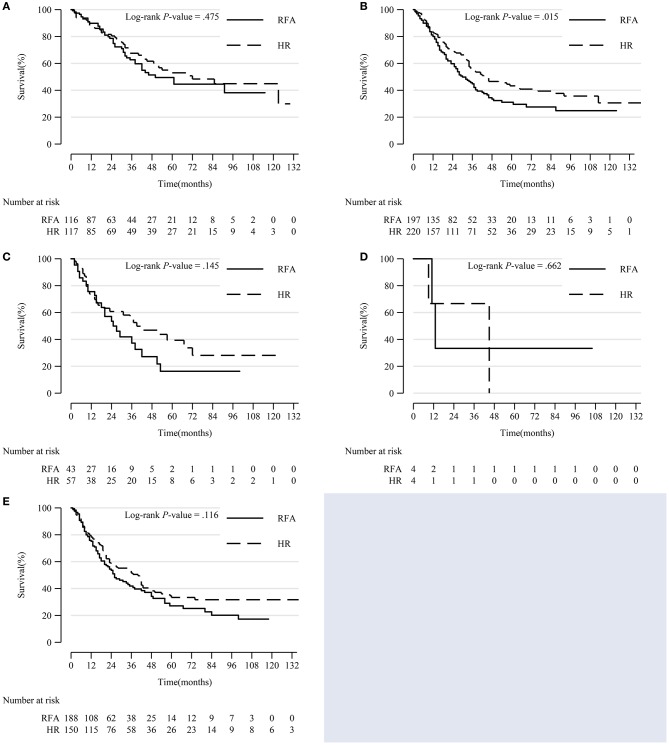
Comparison of Kaplan-Meier survival curves of DSS between the HR and RFA groups stratified by differentiation grade after PSM. Patients treated with HR and RFA had similar survival rates in well **(A)**, poor **(C)**, undifferentiated/anaplastic **(D)**, and unknown **(E)** differentiation subgroups. However, patients treated with HR had a significantly improved survival in moderate differentiation subgroup **(B)**. DSS, disease-specific survival; HR, hepatic resection; RFA, radio-frequency ablation; PSM, propensity score matching.

Within each subgroup of tumor differentiation grade, the survival rate of the HR group was higher than that of the RFA group, but only the difference in the moderate differentiation subgroup was statistically significant (*p* = 0.015) (Figure 3).

**Figure 4:**
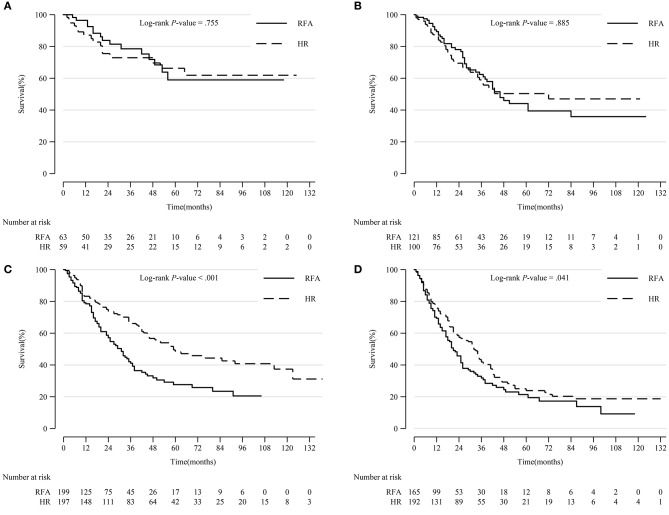
Comparison of Kaplan-Meier survival curves of DSS between the HR and RFA groups stratified by tumor size after PSM. Patients treated with HR had comparable prognoses with those treated with RFA in tumor size 0–2 cm **(A)** and 2–3 cm **(B)** subgroups. However, patients treated with HR had a significantly prolonged survival in tumor size 3–5 cm **(C)** and >5 cm **(D)** subgroups. DSS, disease-specific survival; HR, hepatic resection; RFA, radio-frequency ablation; PSM, propensity score matching.

In patients with tumors sized 3–5 cm and >5 cm, HR was significantly associated with a higher survival rate (*p* < 0.001 and *p* = 0.041, respectively). There was no notable difference in prognosis between patients who underwent HR and RFA for tumors 0–2 and 2–3 cm in diameter (Figure 4).

**Figure 5:**
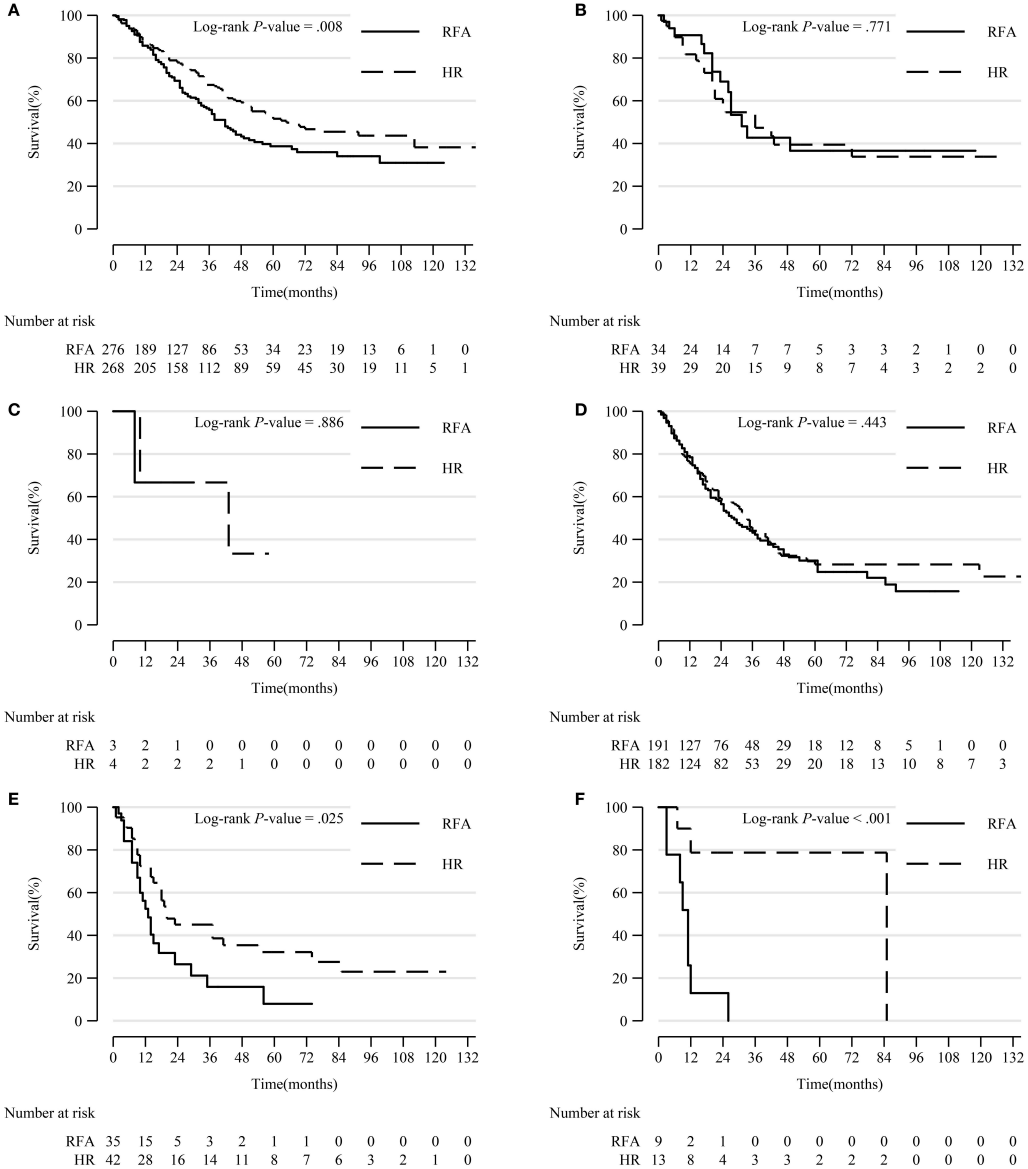
Comparison of Kaplan-Meier survival curves of DSS between the HR and RFA groups stratified by tumor extension after PSM. Patients treated with HR had a significantly better prognosis than those treated with RFA in subgroups of tumor extension 390 **(A)**, 630 **(E)**, and 635 **(F)**. However, patients treated with HR had a comparable survival with those treated with RFA in tumor extension 400 **(B)**, 420 **(C)**, and 440 **(D)** subgroups. DSS, disease-specific survival; HR, hepatic resection; RFA, radio-frequency ablation; PSM, propensity score matching.

In patients with tumor extension 390, 630, and 635, HR led to significantly prolonged survival compared to RFA (*p* = 0.008, *p* = 0.025, and *p* < 0.001, respectively). However, in patients with other tumor extensions, HR resulted in a comparable survival rate with RFA (Figure 5).

## Discussion

In this investigation, we evaluated the efficacies of HR and RFA in patients with multifocal HCC with the presence of big tumor size and vascular invasion. We found that HR was superior to RFA for the treatment of multifocal HCC sized 3–5 cm and larger than 5 cm, while HR had comparable efficacy with RFA for multifocal HCC sized 0–2 and 2–3 cm.

Generally, HR and RFA are recommended as primary therapies for very-early- and early-stage HCC; however, the efficacies of HR and RFA applied to multifocal HCC tumors have not been adequately elucidated. In our study, we adjusted for age, race, marital status, tumor differentiation grade, radiotherapy and chemotherapy, lymph node metastasis, tumor size, distant metastasis, and tumor extension. Tumor extension indicates the status of tumor invasion into both lobes, intrahepatic vascular, gallbladder, the surface of liver parenchyma, and macrovascular (17–21). After the adjustment, significant differences in DSS and OS were observed between the HR and RFA groups; moreover, PSM did not affect the effectiveness of HR on DSS and OS. We found that HR was a more favorable treatment modality for patients with multifocal HCC than RFA, which was consistent with the findings of other studies of non-multifocal HCC with similar tumor size (9, 10, 13, 14, 22, 23).

As a non-invasive treatment, RFA is recommended as the first-line treatment by the American Association for the Study of Liver Disease, European Association for the Study of Liver Disease, and European Society for Medical Oncology for HCC of BCLC stage 0 and A. Recently, the application of RFA has expanded to tumors sized >5 cm, and it is considered to be as favorable as stereotactic body radiotherapy for BCLC advanced-stage HCC, while the application of HR has remained unchanged (24, 25). The efficacy of RFA is affected by tumor size, the number of nodules and the location of the tumors, and it may not be applicable for multifocal tumors in a central location or those invading both lobes (5). Our study proved that RFA was associated with a worse prognosis in most patients as compared with HR after considering multifocality, tumor size and vascular invasion.

According to some studies, RFA and HR have similar efficacies for single tumors smaller than 2 cm without macrovascular invasion or extrahepatic metastasis. Although the sample sizes of these studies were small and the multifocality status of the participants was unknown, they did reveal the potential feasibility of HR for tumors <2 cm (26, 27). In our study which focused on patients with multifocal HCC, we also found HR led to a similar survival outcome as RFA in patients with tumors sized <3 cm; however, HR resulted in a considerable survival advantage over RFA among patients with HCC tumors sized 3–5 and >5 cm. In patients with tumors larger than 3 cm in size but not exceeding 5 cm, those who underwent HR had a superior outcome compared to those who underwent RFA in both the multivariate regression and subgroup analysis, consistent with previous research (13, 14, 22, 28). Moreover, a novel finding of our study was that HR was a valuable treatment modality superior to RFA for multifocal HCC sized larger than 5 cm. In summary, the efficacy of HR maintained robust with increasing tumor size, while the efficacy of RFA declined, which was probably due to that large tumors raise the possibility of excessive distance from the heat source, incomplete coagulative necrosis and undetectable nodules in RFA procedure.

In our study, HR included wedge resection, segmental resection, lobectomy and hepatectomy, because there is no prognostic difference between anatomical and non-anatomical HR for HCC according to published literature (29, 30). For multifocal HCC, a large part of the liver will be excised by HR, resulting in a higher risk of liver function loss, especially in patients with cirrhosis. Hepatic failure is the most severe complication of HR; however, conservative treatment following HR can result in recovery. Studies have argued that HR could result in a long survival time even for HCC in BCLC intermediate-stage, with the presence of distant metastasis and portal vascular invasion, but multifocality was not mentioned (28, 31, 32). Until now, data for survival outcomes related to the treatment of multifocal HCC have been limited. The present study proved that HR is superior to RFA for multifocal HCC tumors, which extended the application of HR for multifocal HCC tumors from early-stage to advanced-stage and validated the expandability of HR (14, 33).

Our multivariate Cox analyses to identify prognostic factors in multifocal HCC patients came to similar conclusions as previous studies that level of AFP and vascular invasion were independent predictors of prognosis. Patients with higher levels of AFP and vascular invasion had significantly worse outcomes than did other patients (7, 17, 30). Vascular invasion as a poor prognostic factor is because vascular invasion is attributed to the recurrence of HCC, which is the primary inducement of postoperative death. According to this study together with other published researches (7, 8, 26), we could conclude vascular invasion was not a contraindication of performing HR in patients with multifocal HCC. Besides, patients with HCC with macrovascular invasion could benefit significantly from HR according to multivariate model as well as subgroup analysis.

A previous study showed that patients younger than 65 years with tumors could benefit more from HR than RFA; however, it did not take into consideration lymph nodes, multifocality, distant metastasis, and vascular invasion (16). By accepting these characteristics into account in our study, we identified a significant disparity between the prognosis associated with HR and RFA. However, age did not affect the effectiveness of HR and RFA in our research.

This study has some limitations. First, many of the factors involved in determining the course of treatment were not captured in the SEER registry, including patient performance, physician recommendations, comorbidities and proximity to treatment providers, which could have biased the treatment allocation (34). Second, the information on details about HR and RFA procedures such as resection margin status, rate of satisfactory ablation, the frequency used and temperature achieved for ablation, as well as complications were all not recorded in the SEER database; however, those were very important confounding factors for assessing the effectiveness of HR and RFA. Finally, the tumor extension does indicate tumor growth, but it does not contain the exact number of nodules, the accurate location of tumors within both lobes, and the comprehensive position of vascular invaded by tumors. Therefore, we could not consider these characteristics within PSM in our analysis.

Despite its limitations, our study adds to our understanding of the efficacy of HR and RFA for multifocal HCC. HR could lead to a promising prognosis in patients with multifocal HCC. Further prospective studies are needed to verify the survival benefits of HR over RFA in patients with multifocal HCC, controlling for the status of resection margin, rate of satisfactory ablation, frequency used for ablation, number of nodules, tumor size, vascular invasion, extrahepatic disease, and simultaneous resection.

## Data Availability Statement

All the data analyzed in this study can be found at https://seer.cancer.gov/.

## Ethics Statement

An ethical review process was not required for this study because the data were obtained from the Surveillance, Epidemiology, and End Results (SEER) database, and we have signed the Data-use Agreement for the SEER 1973–2015 Research Data File.

## Author Contributions

YY contributed to the design of the study, acquisition, analysis, and interpretation of the data, drafting, and critical revision of the article. WZ contributed to the design of the study, acquisition, and analysis of the data. YY and WZ have read and approved the final version of the article, and both agree to be accountable for its content.

### Conflict of Interest

The authors declare that the research was conducted in the absence of any commercial or financial relationships that could be construed as a potential conflict of interest.
